# Pulmonary Artery and Pulmonic Valve Vegetations in a Young Pregnant Filipino with Patent Ductus Arteriosus

**DOI:** 10.1155/2019/8268296

**Published:** 2019-07-09

**Authors:** Valerie R. Ramiro, Jezreel L. Taquiso, Stephanie Martha O. Obillos, Charlene F. Agustin, Jose Donato A. Magno, Eric Oliver D. Sison

**Affiliations:** Section of Cardiology, Department of Medicine, University of the Philippines-Philippine General Hospital, Philippines

## Abstract

**Background:**

Infective endocarditis (IE) involving the pulmonic valve and/or the pulmonary artery is rare. An unrepaired patent ductus arteriosus (PDA) is a risk factor for IE. A previous IE is also a risk factor that predisposes to IE recurrence. Discriminating between IE recurrence and a persistence of a vegetation from a previously treated IE can be difficult. We present the case of a 19-year-old primigravid with an unrepaired PDA and a history of IE treated 7 years prior, with positive blood cultures and vegetations on the pulmonic valve and pulmonary artery seen on transthoracic echocardiogram (TTE).

**Methods and Results:**

On TTE, a small-sized PDA with a Qp : Qs of 1.18 and vegetations on the pulmonic valve and pulmonary artery were documented. Despite the paucity of symptoms, she was empirically treated as culture-negative IE and given 2 weeks of ceftriaxone. Repeat TTE done after 2 weeks only showed a slight decrease in the vegetation size. Due to the paucity of symptoms of infection, lack of growth of the vegetation, and absence of embolic events, the vegetations were deemed to be persistent remnants from the previous IE rather than a recurrent IE. She was advised surgical PDA closure and harvest of vegetations after delivery, but the patient did not consent. The rest of her perinatal course was uneventful.

**Conclusion:**

Persistence of vegetations despite successful medical treatment occurs in some cases and has not been reported to be associated with increased morbidity. Therefore, a follow-up of IE after treatment should be guided by the clinical course and response to therapy as well as the echocardiographic morphology of vegetations over time.

## 1. Introduction

The involvement of the pulmonic valve and the pulmonary artery in infective endocarditis (IE) is rare. Unrepaired congenital heart disease (CHD), including patent ductus arteriosus (PDA), is a risk factor for IE. A previous IE is likewise a significant risk factor that predisposes to IE recurrence. Discriminating between IE recurrence and a persistence of a vegetation from a previously treated IE can be difficult. This paper is aimed at describing a case of pulmonic valve and pulmonary artery vegetations in a pregnant patient with an unrepaired PDA and a distant history of IE treatment. This is the first documented case of pulmonic valve and pulmonary artery vegetation in a patient with a PDA in our institution.

## 2. Case

This is the case of 19-year-old primigravid with an unrepaired PDA who was incidentally found to have vegetations on the pulmonary artery and pulmonic valve on routine prenatal transthoracic echocardiogram (TTE).

She had an unremarkable childhood until 10 years old when she was diagnosed with moderate patent ductus arteriosus, presenting as exertional dyspnea. She was lost to follow-up until 2 years later, at 12 years old, when she was admitted at the emergency room for edema, exertional dyspnea, and orthopnea. On physical examination, she was in respiratory distress, tachypneic with alar flaring, and had bilateral crackles. She was initially treated for community-acquired pneumonia and pulmonary tuberculosis, with initial blood cultures yielding *Streptococcus viridans*. This was treated with Penicillin G. However, despite improvement of pneumonia, she was persistently febrile. Repeat blood cultures were positive for *Pseudomonas putida*. A TTE revealed multiple vegetations at the pulmonary artery and pulmonic valve. The IE was then treated with a 6-week course of intravenous meropenem. Furosemide, digoxin, and enalapril were also initiated for the heart failure. Surgery to close the PDA and remove the vegetations was offered but was not done due to the lack of consent and funds. Serial TTE done during the following year did not show a significant decrease in the size of the pulmonary artery masses. She was again lost to follow-up but continued taking furosemide, digoxin, and enalapril.

**Figure 1:**
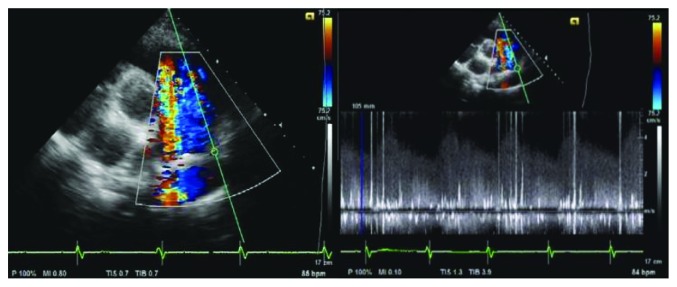
In the short axis view at the level of the aortic valve, there is a retrograde mosaic jet entering the distal pulmonary artery from the posterolateral direction, indicative of patent ductus arteriosus, with the following measurements: PSAX 9 mm, 10 mm (with color); suprasternal 8.1 mm, 1 mm (with color). Continuous flow across the defect is demonstrated on spectral Doppler. Computed Qp : Qs is 1.18.

**Figure 2:**
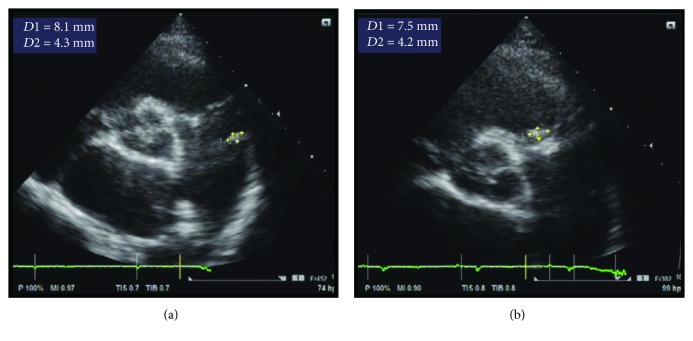
In the short axis view at the level of the aortic valve, there are mobile echogenic vegetations attached to the pulmonary artery (a) measuring 8.1 × 4.3 mm and on the pulmonic valve (b) measuring 7.5 × 4.2 mm.

She returned to our institution for prenatal care. She discontinued taking furosemide, enalapril, and digoxin at the start of pregnancy. Given her history of a congenital heart disease, a TTE was ordered as part of the prenatal work-up. The TTE was done on her 29th week AOG, and it revealed a PDA with a computed Qp : Qs of 1.18 (see Figure 1), as well as vegetations on the pulmonic valve and pulmonary artery (see Figure 2).

Despite the absence of fever and signs of worsening heart failure, she was preemptively admitted and treated empirically for IE with intravenous ceftriaxone. During this period, she did not have fever nor signs of decompensation. Serial blood cultures were negative. On repeat TTE after 2 weeks, there was only a slight decrease in the size of the vegetation and it was treated as a persistent vegetation from her previous bout of IE at 12 years old. She was advised surgery, for closure of the PDA and harvest of vegetations, after delivery, but the patient did not consent. The rest of her perinatal course was uneventful.

## 3. Discussion

According to the European Society of Cardiology (ESC), at present, 0.2 to 4% of all pregnancies in western industrialized countries are complicated by cardiovascular diseases. Maternal heart disease, in general, is now said to be the major cause of maternal death during pregnancy. Because of improved treatment of CHD during childhood, more of these patients reach child-bearing age. In western countries, CHD is the most frequent cardiovascular disease present during pregnancy (75–82%) [1]. In our institution, rheumatic heart disease is still the predominant cardiac complication among pregnant patients followed by CHD [2]. Another study done in our institution showed that among pregnant patients with CHD, PDA (20%) was one of the most common lesions encountered [3]. Patients with small-sized PDA, without Eisenmenger syndrome, may tolerate pregnancy well [1].

An unrepaired PDA is a risk factor for IE, and when it occurs, the vegetation is often on the pulmonary artery side of the PDA [4]. The proposed pathogenesis of IE associated with CHD involves complex interactions among the damaged valvular or mural endocardium exposing the matrix proteins to thromboplastin and tissue factor, formation of a nonbacterial thrombotic endarteritis, and eventual microbial adherence, colonization, and replication [4]. The turbulent blood flow through the aorta and pulmonary artery which causes endothelial injury and subsequent seeding of pathogens onto the injured endothelium is central to the proposed pathogenetic mechanisms for the development of vegetations particularly in patients with PDA [5].

IE involving the pulmonic valve is rare. The estimated incidence of IE associated with PDA was reported to be 1% per year with the decline in recent years attributed to early treatment with routine closure of PDA, improved dental care, and prevalent use of antibiotics both for prophylaxis and for IE treatment [4]. A review done by Knirsch and Nadal in 2011 on IE in CHD presented pooled percentages of distribution of IE according to anatomical localization based on data of retrospective clinical studies. Affectation of the pulmonic valve was 8.8%, the pulmonary artery was 2.3%, and the PDA was 2.7% [6]. A retrospective study done in our institution by Javier et al. reviewed 135 cases of patients with TTE findings consistent with IE from 2004 to 2009. Of these, 91 cases fulfilled Duke's criteria for definite IE. On their review, they found that CHD was the second most common underlying cardiac abnormality (17.5%), 21.1% of which was PDA [7].

There have been several case reports on IE involving either the pulmonary artery or the pulmonic valve, but their outcomes have either been unknown as the patient was lost to follow-up [8] or documented to be resolved by surgical resection or antibiotics [5, 9–11]. In our patient's case, the vegetation persisted despite completion of culture-guided antibiotic therapy during her initial episode of IE at 12 years old. A surgical harvest of the vegetation with concomitant closure of the PDA was not performed at the time as the patient was lost to follow-up.

A previous IE is a significant risk factor that predisposes to IE recurrence. A prospective cohort study by Alagna in 2013 reported a 4.8% rate of repeat IE. A previous IE was one of the associated risk factors, with odds ratio of 2.8 (95% CI 1.6-5.4) [12]. However, in patients with poor documentation or recall of their past medical history, discriminating between IE recurrence and a persistent or residual vegetation from a previously treated IE may be difficult.

There is no strong recommendation for the closure of a PDA to prevent an initial attack especially for small or silent PDA [13]. However, in our patient who had a history of previous IE, a surgical closure of the PDA with concomitant harvest of vegetations was warranted. Unfortunately, the patient did not consent to the procedure despite being advised of the risk of embolization of the vegetation and the risk of Eisenmenger syndrome.

Resolution of the vegetation after antibiotic treatment is one of the indications of successful treatment of IE. However, a 1994 retrospective study by Vuille et al. describing the natural course of vegetations after medical therapy found that out of 41 vegetations, 29 vegetations persisted at the end of treatment (59% of which had no significant change in size, and 52% appeared denser). This led to the conclusion that despite successful medical treatment, persistence of vegetations is still commonly demonstrated echocardiographically and that treatment of IE should be guided by the clinical evolution or response to therapy, rather than the echocardiographic changes of a vegetation [14]. In recent years, this concept is still maintained. The 2010 ESC recommendations for the practice of echocardiography in IE also acknowledge the difficulty of interpreting an unchanged or reduced vegetation size with antibiotic treatment. However, the progressive growth of a vegetation and increasing valvular regurgitation foreshadow poor prognosis as they are risk factors for embolic events and morbidity [15]. The mere persistence of a vegetation despite paucity of supportive evidence for IE, such as symptoms or positive blood cultures, is not associated with increased morbidity [16]. Therefore, a follow-up of IE after treatment should be guided mainly by the clinical course and response to therapy and not only by the change over time of the echocardiographic morphology of vegetations.
